# A case report on H syndrome among patients with diabetes mellitus

**DOI:** 10.6026/9732063002001295

**Published:** 2024-10-31

**Authors:** Krisha Jain, Rama Krishna Srirangam, Rishabh Jain, Alekhya Emandi, Saurabh Mutha, Ravi Sangoi, Sai Vanniappan Meenakshi Sundaram, Mayur Wanjari

**Affiliations:** 1 Department of Paediatrics, Government Medical College and General Hospital, Baramati, Pune, Maharashtra, India; 2 Department of Acute Medicine, Queen Elizabeth Hospital, Birmingham, UK; 3 Medicine Senior House Officer (SHO), Cumberland Infirmary, North Cumbria Integrated Care NHS Foundation Trust, United Kingdom; 4 Department of Internal Medicine, ASRAMS, Eluru, India; 5 Department of General Medicine, Rajah Muthiah Medical College, Chidambaram, Tamil Nadu, India; 6 Department of Research, Datta Meghe Institute of Higher Education & Research (DMIHER), Sawangi, Maharashtra, India

**Keywords:** Autosomal recessive, diabetes mellitus, h syndrome, hyperpigmentation, lymphadenopathy

## Abstract

This case describes an 8-year-old female with H syndrome who came in with diabetes mellitus (DM), highly stressing the very striking features of the clinical presentation and possible challenges that might intervene in its treatment. H syndrome is an autosomal recessive disorder with scarce information on complications and possible interventions. As H syndrome is extremely rare, there are very few treatment experiences and most of them are unsatisfactory.

## Background:

H syndrome is an autosomal recessive condition which is rare and described by several clinical features and other systemic manifestations which include cutaneous hyperpigmentation, hypertrichosis, hepatosplenomegaly, hearing loss, cardiac defects, hypogonadism, very high sugar levels, low height and hallux valgus [1]. The term "H Syndrome" derived from the data that patients in the study conducted for the time about this syndrome were presented with symptoms all stating with letter "H" [5]. It is genodermatosis caused by the mutations in the gene named SLC29A3. This gene encodes a transporter called the human equilibrated nucleoside transporter 3 (hENT3). It localizes to cell organelles such as endosomes, lysosomes and mitochondria [2, 3] The distinguishing characteristic of this condition excluding cutaneous hyperpigmentation and hypertrichosis is the thickening and hardening of skin typically involving the inner part of thighs and shin bony area with clear visible sparing of the skin over the knee joint. The broad spectrum of histiocytosis-lymphadenopathy plus syndrome under the OMIM classification has #602782. Males with H syndrome are described with symptoms such as scrotal masses, gynecomastia, and azoospermia [4]. Other features described include varicose veins and joint deformities (hallux valgus and fixed flexion contractures of interphalangeal joints) [6]. According to the present data available, there is no specific and conclusive treatment of this disorder which makes it even more salient to recognize this condition; thus, neglecting the unnecessary interference for treating cutaneous manifestations. In various studies, during management genetic counselling is said to play a major role [7]. The treatment is only supportive and palliative i.e. focuses on treating the symptoms. Here we present a case of H syndrome with diabetes mellitus in 8-year-old female who presented with hypertrichosis and hyperpigmentation of lower limbs.

## Case presentation:

An 8-year-old female born out of a consanguineous marriage presented to the outpatient department (OPD) in our hospital with chief complaints of hypertrichosis and hyperpigmentation (Figure 1 - see PDF) of lower extremities and trunk. The patient was conscious and well oriented to time, place and person. Developmental milestones achieved were normal. The patient also had hearing loss but was not using any aid for the same. She has no history of weight loss or any radiation exposure. There was no family history of any similar skin damage. The patient has a short stature and has normal weight as per BMI. Her height was 98cm (percentile) and 28kg weight was (percentile). Her pulse rate was 70/min and blood pressure was 90/60 mmHg. She had pale skin. On general physical examination, she showed low height for weight, hepatosplenomegaly, pallor and webbing of neck, mild proptosis and normal genitalia. General examination also showed severely thick, edematous and hairy lower limbs. Icterus and Lymphadenopathy were absent. The skeletal examination was normal. On mucocutaneous examination well defined, bilaterally symmetrical hyperpigmented, indurated plaques with marked hypertrichosis were present over the medial aspect of thighs and legs. Similar lesions were present bilaterally over the gluteal region. Although the knees were spared. Auditory evaluation revealed bilateral sensorineural hearing loss.

The patient appeared in stable condition and had normal vital signs. Laboratory investigations (Table 1) included CBC which suggested microcytic anemia with hemoglobin levels of 10.9g/dl. WBCs were over 11020/dl and platelet count of over 4.21lakhs/dl. Laboratory investigations also revealed elevated ESR (erythrocyte sedimentation rate) and CRP. Her blood sugar levels were high with HbA1C as high as 16.30, suggestive of Type 1 Diabetes Mellitus. Creatinine levels were 0.37 and the ratio AST/ALT was 11/20. Liver function tests and thyroid profile were normal. Chest X-ray was normal. Antinuclear antibody was negative. An USG abdomen revealed hepatosplenomegaly. Chest X ray showed no significant findings. Microbiological tests for infectious diseases did not provide any significant findings. As the diagnosis of H syndrome was confirmed, the patient's therapeutic and management plan was initiated. This included Insulin therapy to treat Diabetes Mellitus. Anti-hypertensive medicines were also started. Patients were also advised to take pancreatic enzyme supplements. The patient's response and the compliance with the therapy were monitored closely. Even minor findings have been noted. Treatment was provided as per the manifestation observed. As there is no specific treatment for this condition, supportive measures were taken up. Also, parents were advised to give genetic counselling to discuss inheritance patterns and the possible implications for future pregnancies. During follow up, all relevant tests were performed and levels were checked.

## Discussion:

H syndrome is an autosomal recessive disorder caused due to biallelic mutation in SLC29A3 gene that encodes hENT3. The human equilibrate nucleoside transporter (hENT3) facilitates passive sodium-independent movement of nucleosides, thereby facilitating cells that lack de novo synthesis to rely on salvage pathway [8] [9]. It is a novel form of histiocytosis involving multiple organ systems. Most common features include cutaneous manifestations, hypertrichosis, hyperglycaemia (diabetes mellitus), hearing loss, short stature, hepatosplenomegaly, *etc.* [1]. Cutaneous manifestations like hyperpigmentation along with thickening which is sclerodermatous and hypertrichosis mainly on the lower limbs is the common finding in about 68% of patients. These findings are seen in this case as well [8]. A skin biopsy may reveal characteristic findings which may help in diagnosis of H syndrome and thereby help in differentiating from other conditions having similar manifestations. Features like fibrosis of the dermal layer, aggregates of lymphocytes and numerous CD68+, CD163+, S100‐positive, and CD1a‐negative dermal histiocytosis are obtained by Histopathologic examination. These features are used to differentiate H syndrome from various similar conditions like morphea, scleroderma and POEMS syndrome which may clinically seem identical [10]. The third most common symptoms in patients with H syndrome is bilateral sensorineural hearing loss. The onset of hearing loss could either be progressive in nature or could be sudden, maybe congenital and range from mild to severe. Mitochondria are a center for energy production in a cell and are a major part of oxidative phosphorylation. As cochlear activity consumes substantial energy, mitochondrial mutations would cause inefficiencies in producing energy, resulting in deafness. In H syndrome, there is a disturbance in the mitochondrial metabolic pathway due to mutation. One of the features of this is progressive hearing loss without the failure of the vestibular system [11]. This defect in mitochondria due to mutation is thought to be the cause for deafness in some H syndrome patients [12]. One of the symptoms of H syndrome is insulin dependent diabetes mellitus. As in our case we can see the patient is hyperglycaemic with HbA1C over 16, there are few studies done to establish as to why SLC29A3 gene causes Type 1 DM. The availability of nucleosides in the cytoplasm is an important requirement for most of the pathways in a cell, particularly the salvage pathway and generation of nucleotides like adenosine and guanosine triphosphates for metabolism of cellular energy and transduction of signal. As per one study on Drosophila melanogaster, there is evidence that suggests that the Drosophila ortholog of this protein interacts with the signaling pathway of insulin. The mutations seen in the equilibrate nucleoside transporter 3 protein are therefore said to be accompanied by a genetic syndrome of insulin-dependent DM [13]. Pigmentary hypertrichosis is a usual finding in patients with H syndrome. However, hypertrichosis is growth of hair in excessive amount and thickness on any part of the body. If hypertrichosis occurs as a component of any syndrome, the management must be multidisciplinary which would address the systemic manifestations [14]. However, the exact cellular localization of hENT3, endosomal/lysosomal or mitochondrial, and its function with relation to the H syndrome is still unclear [9]. Considering how rare H syndrome is, the diagnosis might be overlooked, which could be harmful to the patient as it is a multi-systemic disorder involving heart, blood, kidneys, endocrine, *etc.*, [1]. Genetic testing establishes diagnosis and aids in future counselling [6]. Considering the number of cases discovered till date, and its overlapping symptoms with other systemic diseases, this report highlights the need for creating awareness among physicians for early detection of this syndrome [4]. Since there is no elusive treatment available for H syndrome, more research on this condition is exceedingly needed. However, a few studies suggest Tocilizumab should be considered as the first choice in treatment, mainly to control the autoinflammatory component of the disease. It can be started as early as required to avoid further complications [15, 16]. A better understanding of the pathogenesis of H syndrome as an autoinflammatory syndrome has improved patients' outcomes [17].

## Conclusion:

H syndrome is an autosomal recessive disorder that is not seen frequently. It occurs due to genetic mutation in SLC29A3 gene with clinical features such as hyperpigmentation, hypertrichosis, hearing loss, hyperglycemia, *etc.* All these features have uniqueness in the presentation of each case. This case report highlights clinical presentation and multi-systemic involvement of a patient with H syndrome.

## Figures and Tables

**Table 1:** Laboratory test report

**Investigation**	**Result**	**Reference**
Hemoglobin	10.9g/dl	11.5-14 g/dl
WBC Total Count	11020/ccm	5000-19000/mm3
Platelet Count	4,21,000	1,50,000-4,50,000
HbA1C	16.30%	Under 8%
Antinuclear Antibody	Negative	
